# Tumor heterogeneity in neoplasms of breast, colon, and skin

**DOI:** 10.1186/1756-0500-3-321

**Published:** 2010-11-25

**Authors:** Jian Li, Kai Wang, Thomas Dyrsø Jensen, Shengting Li, Lars Bolund, Carsten Wiuf

**Affiliations:** 1 Institute of Human Genetics, Aarhus University, DK-8000 Aarhus, Denmark; 2 BiRC - Bioinformatics Research Centre, Aarhus University, DK-8000 Aarhus, Denmark; 3 BGI-Shenzhen, Shenzhen 518083, China

## Abstract

**Background:**

Different cell subpopulations in a single tumor may show diverse capacities for growth, differentiation, metastasis formation, and sensitivity to treatments. Thus, heterogeneity is an important feature of tumors. However, due to limitations in experimental and analytical techniques, tumor heterogeneity has rarely been studied in detail.

**Presentation of the hypothesis:**

Different tumor types have different heterogeneity patterns, thus heterogeneity could be a characteristic feature of a particular tumor type.

**Testing the hypothesis:**

We applied our previously published mathematical heterogeneity model to decipher tumor heterogeneity through the analysis of genetic copy number aberrations revealed by array CGH data for tumors of three different tissues: breast, colon, and skin. The model estimates the number of subpopulations present in each tumor. The analysis confirms that different tumor types have different heterogeneity patterns. Computationally derived genomic copy number profiles from each subpopulation have also been analyzed and discussed with reference to the multiple hypothetical relationships between subpopulations in origin-related samples.

**Implications of the hypothesis:**

Our observations imply that tumor heterogeneity could be seen as an independent parameter for determining the characteristics of tumors. In the context of more comprehensive usage of array CGH or genome sequencing in a clinical setting our study provides a new way to realize the full potential of tumor genetic analysis.

## Background

Tumor heterogeneity is defined as the simultaneous presence of multiple clonal subpopulations of tumor cells within a single neoplasm [1]. The concept of tumor heterogeneity implies the important fact that all of the components actually both constitute and contribute to a tumor society. Thus heterogeneity becomes an important issue in cancer research since different subpopulations may show different capacities for growth, differentiation, metastasis formation, as well as sensitivity to radiation and chemotherapeutic treatments [2,3].

To date, some attempts have been made to decipher tumor heterogeneity on the basis of the genetic profiles of a few tumor subpopulations by using a combination of laser-microdissection, PCR amplification, and comparative genomic hybridization (CGH) or array CGH [4,5]. These studies revealed DNA copy number profiles for individual subpopulations from specific sampling sites. This may lead to a unilateral, rather than a global view of the tumor society. Additionally, laser microdissection is a time-consuming and labor-intensive approach and amplification of tiny amounts of tumor cells may cause bias in the subsequent analysis. Recently, SNP [6] and CGH array [7] analyses have been used to describe tumor heterogeneity. However, the developmental process of tumor heterogeneity remains unclear.

We developed a mathematical method to estimate tumor subpopulations and their corresponding percentages in tumors through the analysis of array CGH data [8]. This approach provides a novel tool to reveal different cell subpopulations and to identify the heterogeneous population structure of a tumor.

Breast cancer, colon cancer and skin squamous cell carcinoma (SCC) are among the most common malignant tumors worldwide [9]. There is overwhelming evidence that breast cancer is a heterogeneous disease [1,10]. Unlike breast cancer, colon cancer is thought to be more homogeneous with a single dominant pathway and histological presentation during tumor progression [11]. SCC and keratoacanthoma (KA) have similar morphological features [12]. However, SCC is malignant and metastatic, requiring aggressive treatments, whereas KA regresses spontaneously after a couple of months as a benign neoplasm [12]. The heterogeneity of SCC and KA has rarely been studied. In the present study, a multiple-type tumor collection with a relatively large sample size allowed us to explore some characteristics of tumor heterogeneity.

### Presentation of the hypothesis

Through the analysis of array CGH data using our established heterogeneity model, we deciphered DNA copy number profiles for the major subpopulations in each tumor. Significant differences of tumor heterogeneity between different tumors suggest that tumor heterogeneity could be a characteristic feature and a potential clinical parameter for characterizing tumors. The relationship between subpopulations in origin-related samples, such as primary tumor, lymph node and distant metastases could give clues to the understanding of tumor development and its prevention.

## Methods

### Testing the hypothesis

#### Tumor samples

Total 268 tumor samples have been analyzed in this study, in which 82 were from breast cancer, 60 from colorectal cancer and 126 from skin tumors. All the samples were fresh frozen after surgery.

#### Breast cancer

Eighty-two breast cancer samples were from 49 patients: 29 pairs of primary breast tumors and their matched axillary lymph node (ALN) metastases, 10 primary breast tumors without ALN metastases at diagnosis and 10 primary breast tumors with ALN metastasis detected (these metastases were not available for this study). Additionally, there were four "normal" breast cancer tissues biopsies that were collected far from the primary site at least 2 centimeters away from the primary site, and regarded as normal by the pathologist. In the present study, we abbreviate primary tumor, axillary lymph node metastasis and "normal" breast tissues as T, M and N, respectively. The samples were diagnosed as high-risk breast carcinomas at the Copenhagen University Hospital during the period between February 2004 and September 2005. The project was approved by the Scientific and Ethical Committee of the Copenhagen and Frederiksberg Municipalities (KF 01-069/03) [13]. The results of breast cancers analyzed by array CGH have been previously published [8,13,14]

#### Colon cancer

Sixty colon cancer samples consisting of 40 samples with low microsatellite instability (MSI-Low) and 20 samples with high microsatellite instability (MSI-High) were studied. All samples were diagnosed as adenocarcinomas of the colon or of the rectum. The samples were from a prospective cohort of colorectal cancer patients, initiated in 2002 by the Danish Colorectal Cancer Group South at Vejle Hospital, Denmark. The project was approved by the science ethical committee and given the number VF 20040109 [15].

#### Skin tumor

The skin samples consist of 102 KAs and 24 SCCs from 126 patients. The samples were collected in Norway, except for 11 samples which are from England (Li J et al unpublished data). This project was approved by the "Regional committee for medical research ethics Sothern-Norway (REK Sør)". The project number is S-06158.

### Array CGH

Array CGH was performed as previously described [14]. Briefly, genomic DNA was isolated from the fresh frozen samples, labeled and cohybridized with normal reference DNA. The arrays used as target for the hybridization contained elements produced from bacterial artificial chromosome (BAC) clones covering the whole genome with a resolution of 1 Mega base (Mb). The clones were obtained from the Wellcome Trust Sanger Institute with annotation based on the 38/36 version of the clone information released by Ensembl. Intensities were extracted and normalized as described previously [14].

### Heterogeneity model

To estimate tumor heterogeneity, we developed a mathematical method and validated the method by simulation and an independent experiments [8]; here we give a brief description of the heterogeneity model. This method contains two steps: **Step one **establishes a model representing the relationship between the experimental data and the exact underlying copy number;

(1) $$\log(z_{ij})\sim N(\alpha \log(C_{i})+\beta ,\sigma^{2}),$$

where z*_ij _*is the ratio of clone *j *in segment *i *and *C_i _*is the theoretical copy numbers ratio of the test sample against the reference sample. The parameters *α *and *β *are constants justified from calibration experiments [8] and appear to be sample independent (but likely platform specific). We extend the *C_i _*in equation (1) in the following way:

(2) $${\bar{C}}_{i}=\sum_{k=0}^{K}p_{k}C_{ik},$$

where *K *is the number of subpopulations, ∑_*k *_*p*_k = 1 _is the percentage of the *k*th subpopulation, _k _= 1, and *C_ik _*≥ 0 is the copy number in the *k*th subpopulation relatively to the copy number of the test sample. The subpopulations are denoted *P_0_*, *P_1_*, ..., *P_k_*, such that the first subpopulation *P_0 _*is assumed to consist of cells without copy number aberration.

**Step two **is a statistical method to infer the number of dominant subpopulations and their copy number profiles from CGH array data. First, the copy number profile and the percentage of cells in each subpopulation are estimated assuming a fixed number of subpopulations, *K*. Second, it is statistically assessed which number of subpopulations fits the data best. The method assumes a model of sequential tumor evolution in which each subpopulation evolves from the previous population by the introduction of new aberrations, or by making aberrations in the previous population more aggravated. In our previous study, the robustness of the statistical model has been validated by both bioinformatic stimulation and independent experiments [8].

### Unsupervised clustering

An unsupervised hierarchical clustering analysis was applied to analyze the similarity of genomic profiles across subpopulations by using the Cluster 3.0 software [16]. The Pearson correlation algorithm was employed for similarity metric calculation. Complete linkage clustering was chosen to organize subpopulations in a tree structure. TreeView software was utilized for visualization of the cluster analysis result [16].

### Statistical analysis

Statistical analysis was performed using the R 2.10.1 package.

## Results

### Heterogeneity in different types of tumors

We applied our heterogeneity model to estimate the major subpopulations in all samples using the array CGH profiles. A summary of the heterogeneity results for the different types of tumors is presented in Table 1. All breast cancer samples contain at least one aberrant subpopulation. Nearly half of the breast cancers have more than 2 aberrant subpopulations. For the colon cancer samples, most of them were classified into the group containing two subpopulations of which one is "normal" including non-tumor cells. Thirty percent of the MSI-High colon cancers have only the "normal" population, whereas this is the case in only 7.5% of the MSI-Low colon cancers. In the two categories of skin samples, ~60% of the KAs (benign neoplasms) have one subpopulation, i.e., a solo "normal" subpopulation. In contrast, only ~17% of the SCCs (malignant counterpart of KA) present only one "normal" subpopulation. Only 4.9% of the KAs have more than two subpopulations, while this is the case in 29% of the SCCs.

**Table 1:** The distribution of subpopulations in three types of tumors

	sub_1	sub_2	sub_3	sub_4	Homogeneity	Heterogeneity	Total
**Breast**							82

**Breast (T)**	0	25 (*51.02%*)	20 (*40.82%*)	4 (*8.16%*)	25 *(51.02%)*	24 *(48.98%)*	49
**Breast (M)**	0	16 (*55.17%*)	10 (*34.48%*)	3 (*10.34%*)	16 *(55.17%)*	13 *(44.83%)*	29
**Breast (N)**	0	2 (*50%*)	1 (*25%*)	1 (*25%*)	2 *(50%)*	2 *(50%)*	4

**Colon**							60

**Colon (MSI-High)**	6 (*30%*)	12 (*60%*)	2 (*10%*)	0	18 *(90%)*	2 *(10%)*	20
**Colon (MSI-Low)**	3 (*7.5%*)	30 (*75%*)	7 (*17.5%*)	0	33 *(82.5%)*	7 *(17.5%)*	40

**Skin**							126

**Skin (KA)**	61 *(59.8%)*	36 (*35.29%*)	3 *(2.94%)*	2 *(1.96%)*	97 *(95.10%)*	5 *(4.90%)*	102
**Skin (SCC)**	4 *(16.67%)*	13 *(54.17%)*	6 *(25%)*	1 *(4.17%)*	17 *(70.83%)*	7 *(29.17%)*	24
							

In the top of the table, "1", "2", "3" and "4" are related to the number of total subpopulations. "Homogeneity" shows the number of the samples that contain total 1 or 2 subpopulations. "Heterogeneity" displays the number of the samples that have 3 or 4 subpopulations. The corresponding number of the subpopulations and its percentage (parentheses) in each type of tumor are presented, respectively. In breast cancers, "T", "M" and "N" are abbreviated for primary tumor, axillary lymph node metastasis and "normal" breast tissues, respectively.

In our study, the samples containing one or two subpopulations are considered to be homogeneous, whereas those containing more than two subpopulations are considered to be heterogeneous. We compared the number of homogeneous and heterogeneous samples in different groups of tumor samples (Table 2). The breast samples are more heterogeneous than the colon and KA samples. Colon cancer is more heterogeneous than KA but not significantly different from SCC. Notably, the KA samples are significantly more homogenous than the SCC samples. In colon cancers, there is no significant difference in heterogeneity between MSI-High and MSI-Low subtypes. Neither is there a significant difference in heterogeneity between breast primary cancers and lymph node metastases. Taken together, in our study, breast cancers are more heterogeneous than the other types of tumors whereas benign KAs have the lowest heterogeneity.

**Table 2:** Comparison of tumor heterogeneity between different types of tumors

Comparison	P-value	Comparison	P-value
**Breast/Colon**	0.0001	**Colon/SCC**	0.135
**Breast/SCC**	0.114	**Colon/KA**	0.027
**Breast/KA**	0.0001	**KA/SCC**	0.0001
**Breast (T)/Breast (M)**	0.723	**MSI-High/MSI-Low**	0.443
			

The Chi-square test was used to test the significance of the difference in heterogeneity between the groups. (T) means primary tumor, and (M) means lymph node metastasis. The corresponding p-values are presented.

### Genomic alterations in the development of subpopulation heterogeneity

**Figure 1:**
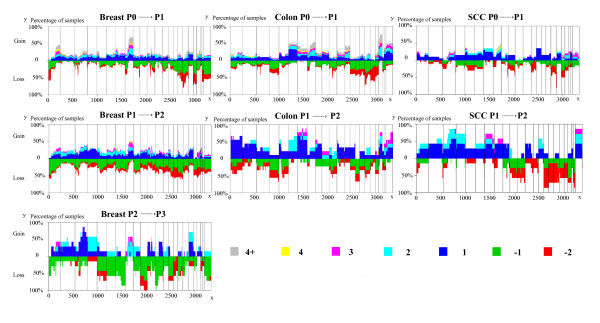
**The development of P0 P1, P1 P2, and P2 P3 subpopulations**. The developments of copy number profiles are divided into three groups if available. From top to bottom, the change of P0 P1 subpopulations, P1 P2 subpopulations, and P2 P3 subpopulations for breast (left), colon (center), and SCC (right) tumor samples. The heights of the vertical lines represent the percentage of samples in which the corresponding clones have DNA copy number alterations. The index of clone is given at the bottom and ordered by genomic position (x-axis). The solid vertical bars demarcate the chromosomes. The different copy number alterations were represented by colors in the lower right corner.

Figure 1 presents the plots of all copy number changes between the consecutive subpopulations within each sample. It illustrates that the developments of P0 P1 subpopulation (the first subpopulation harboring genomic aberrations), P1 P2 subpopulation, and P2 P3 subpopulation (if available), respectively. It should be noted that 9 out of 60 colorectal samples were classified as only "normal" with no corresponding P1 subpopulation detectable. Thus the maximum height of the loss in the plot colon P0 P1 of Figure 1, corresponds to 51 subpopulations instead of 60. No colon sample had a P3 subpopulation. In the case of SCC, only two samples in each group had a P3 subpopulation. Thus a plot of the development of P2 P3 was not considered meaningful. The recurrent genomic aberrations in the P1 subpopulation in more than 50% of the samples for each cancer type have been displayed in additional file 1. The most common aberrations in the benign KA tumors are also shown although they are only seen in 40% of the tumors with a P1 subpopulation. A loss on chromosome 17 is recurrently seen in breast cancer, colon cancer and skin squamous cell cancer. A gain on chromosome 8 is commonly shown in breast cancer and colon cancer. The recurrent alterations in the development of P1 P2, and P2 P3 in more than 50% of the samples for each type tumor have been displayed in additional file 2.

### The relationship of subpopulations in primary tumors, metastases and "normal" tissues

**Figure 2:**
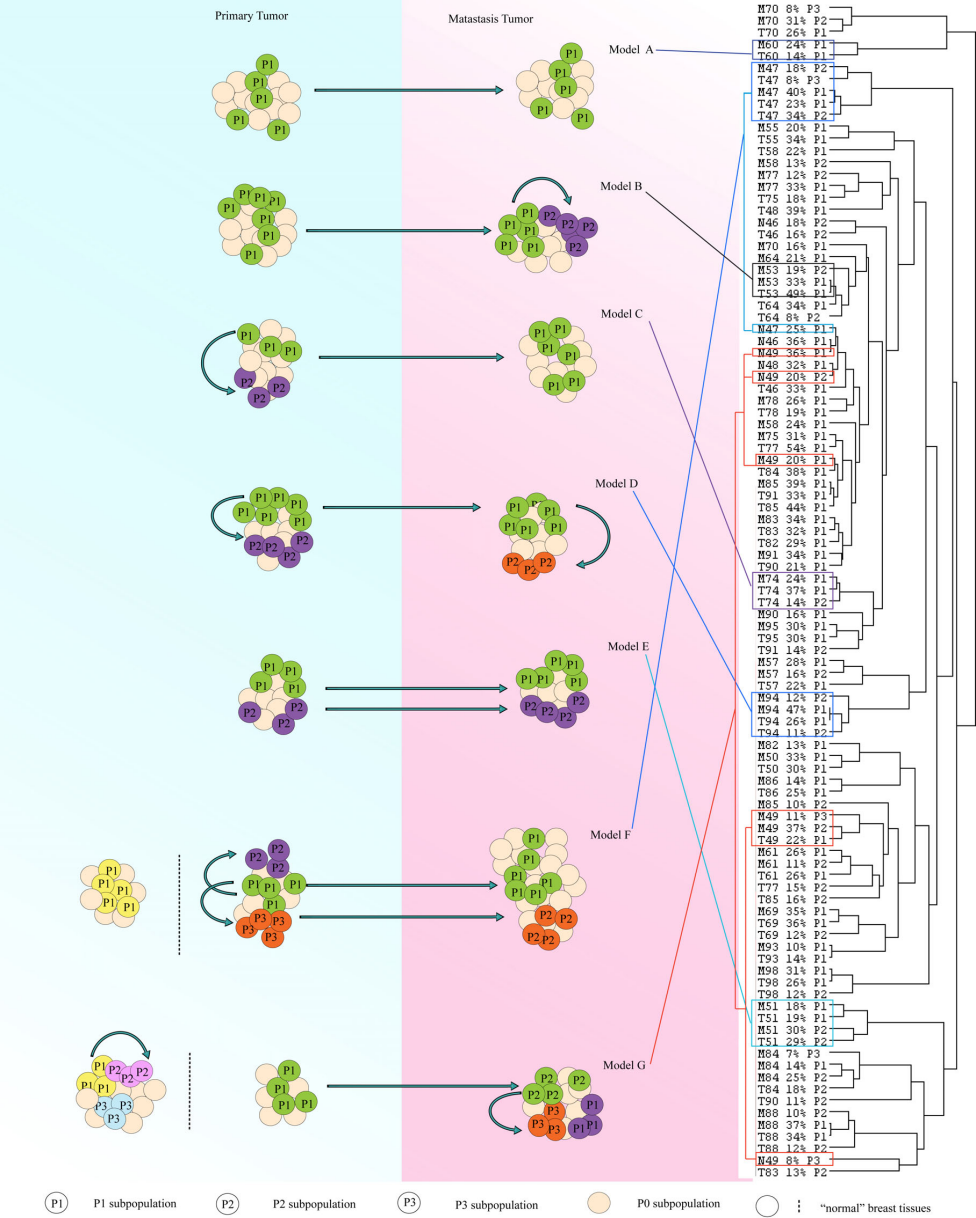
**The relationship between subpopulations in origin-related samples**. Twenty-nine pairs of breast primary tumors (T), lymph node metastases (M) and four "normal" breast tissue samples (N) were analyzed by unsupervised clustering. The similarity of genomic profiles of subpopulations between samples is displayed by clustering (right). The corresponding hypothetical models are shown in the left. The subpopulations presenting the same color denotes they have a close relationship. For more explanations see the text.

In many breast cancer cases, we collected the primary tumors and their matched lymph node metastases. In addition four breast tissue samples were collected from an area at least 2 centimeters from the primary tumor. This tissue was regarded as normal by the pathologist. These origin-related samples allow us to investigate the developmental and evolutionary relationships among the subpopulations in both intra-tumor and inter-tumor comparisons. All origin-related breast cancer samples underwent heterogeneity model analysis, and the estimated subpopulations in each sample were derived. Subsequently, unsupervised clustering analysis was carried out to explore the relationships between subpopulations in all origin-related breast cancer samples. Complex and multiple relationships of subpopulations in tumorigenesis are revealed in Figure 2. Except for the cases 82, 58, 75, 77 and 90, the pair-matched samples present the closest relationships between the corresponding subpopulations in the primary tumors and the metastases. Based on the clustering result, we suggest seven hypothetical models to explain the relationships between subpopulations in origin-related samples (Figure 2). For example, the convergence of T60-P1 and M60-P1 revealed the simple relationship that metastatic cell subpopulation P1 was derived from its primary tumor cell subpopulation P1 (Model A). In Model B, subpopulation P1 in metastasis (M53) could be derived from the subpopulation in the primary tumor (T53) and subsequently develops a new subpopulation (M53-P2) in the metastasis. T74 contains two aberrant subpopulations (P1 and P2), and T74-P1 presents a direct correlation with M74-P1 (Model C). Also, one subpopulation in the primary tumor could directly give rise to the P1 subpopulation in metastasis but then both T-P1 and M-P1 could develop new P2 subpopulations in the primary tumor and in the metastasis, respectively (Model D). In some cases, the subpopulations of the metastases could be derived from multiple subpopulations in the primary tumor. For instance, T51-P1 is clustered with M51-P1, and T51-P2 is clustered with M51-P2 (Model E). There are also some complex models such as Model F and Model G, in which the subpopulations in the "normal" tissue samples do not show any relationships with the tumor subpopulations. In Model G some subpopulations are remarkably unrelated to the next (e.g. N49-P3 and M49-P1).

In contrast, all the N-P1 samples cluster and in case N46-P1 and N46-P2 showed correlations with T46-P1 and T46-P2, respectively. The above observations suggest that the relationship between aberrant subpopulations in "normal" breast tissue samples and the corresponding tumors varies from case to case and that more has to be learnt before the predictive value of aberrant subpopulations can be fully exploited.

### Implications of the hypothesis

All cancers can be regarded as a heterogeneous cell community that originates from a single progenitor and becomes diverse through the acquisition of accumulated alterations in the course of neoplastic evolution [17]. Cancer development is based on two processes: The continuous acquisition of heritable genetic variation in individual cells resulting in more or less random phenotypic change; Natural selection acting on the resultant phenotypic diversity [18]. Most aberrant cells are exterminated and cells with the capability to proliferate and survive more effectively than their neighbors get a selective advantage. Therefore, heterogeneity is a natural feature for cancers.

### The relationship between tumor type and tumor heterogeneity

In this study, tumor heterogeneity seems to be a parameter showing characteristics for different types of neoplasms, suggesting an association with tumor behavior. In general, the breast, colon and skin tumors studied (including benign and malignant counterparts from epidermal tissue) present significantly different degrees of heterogeneity. The breast cancers are more heterogeneous than the other types of tumors. Benign karatoacanthomas have the lowest heterogeneity. This observation suggests that heterogeneity is a tumor-specific feature. The different levels in heterogeneity could reflect different selection processes. Alternatively, the differences in heterogeneity could be due to the bias in sampling. For example, the skin tumors should be detected earlier in their development than breast tumors. More importantly, the breast cancer samples in our study are collected from high risk patients [19]. The higher heterogeneity found in breast cancer could thus reflect the "high risk" feature of this sampling. In the future, more stringently ascertained cancer samples are needed for determining the relative heterogeneity feature of the different types of cancers.

In the colon cancers, the samples with MSI high level are generally assigned lower N and M stages (frequently N0M0), and rarely metastasize to liver compared with the ones with MSI low level [20]. However, the reason that renders MSI-High less aggressive than MSI-Low remains unclear. In this study, we found a non-significant tendency towards lower heterogeneity levels in MSI-High samples than in MSI-Low samples, providing one possible explanation for the above phenomenon.

Keratoacanthoma (KA) is a benign keratinocytic neoplasm that can spontaneously regress [12]. However, KAs share many features such as infiltration and cytological atypia with squamous cell carcinomas (SCCs), which compromises diagnosis and patient management [12]. To date, no simple pathological criteria can give a satisfactory discrimination between KAs and SCCs [12]. In this study, we observed that SCCs showed much higher heterogeneity than KAs. As a benign tumor, more than half KAs have only one subpopulation without any numerical genomic aberrations. Furthermore, less than 5% of the KAs have more than 3 subpopulations in the tumors. By contrast, 83.33% of the SCCs have more than 1 subpopulation, and 29.17% of the SCCs have more than 2 subpopulations.

SCCs might have poorer genetic integrity and produce more aberrant subpopulations than their benign counterpart (KAs), even though the morphological and pathological features are quite similar between the two kinds of epidermal neoplasms [12]. The fact that there are more subpopulations in SCCs than in KAs might account for the more aggressive behavior of SCCs, including metastatic potential. It is conceivable that a tumor with higher number of subpopulations have a greater chance to produce a population that is capable of successful establishment of metastases through the selective process.

### The development process of P0 P1, P1 P2, and P2 P3

As illustrated in Figure 1, characteristic genomic aberrations are already established in the P1 subpopulation of each tumor. The recurrent aberrations such as the loss in chromosome 17 shown in breast cancer, colon cancer and skin squamous cell cancer, and the gain in chromosome 8 found in breast cancer and colon cancer suggest common characters of all malignant tumors. The rest of the recurrent genomic aberrations in the P1 subpopulations might represent unique features related to the specific tumor type. The difference between P1, P2 and P3 within each type of tumor are not dramatic, suggesting that the development of tumor heterogeneity is a continuous and accumulative process. In the context of the evolutionary course of the tumor, the P1 subpopulation already presents basic and essential genomic aberrations for the given type of tumor and thus should be diagnostically and prognostically informative.

### The relationship between subpopulations in primary tumor, metastasis and "normal" tissues

Elucidation of the changes in heterogeneity between metastases and their original primary tumors should deepen our understanding of the mechanisms of metastasis. As a result from the selection in a new microenvironment of the second organ where metastases are established, subpopulations from the primary tumor could decline and even be wiped out, while new subpopulations could emerge in the newly established metastasis. Tracking the alterations in the subpopulations might help to identify metastatic favoring features.

Our previous analysis of the close relationship between breast cancer primary tumors (T) and their lymph node metastases (M) was based on the analysis of whole tumor genomic profiles [14]. The decomposition of all subpopulations of T and M should allow us to explore the reason for the remarkable similarity in genomic profiles between the primary and metastatic tumor. This may also provide clues in search for metastasis mechanisms. In 29 pairs of breast cancer samples, we found that the percentage and profile of subpopulations did not change largely in the metastatic process [8]. However, lymph node metastases and their matched primary tumors present multiple subpopulation relationships. In some cases (Model C), the metastatic cell subpopulation P1 is derived from its primary tumor cell subpopulation P1, but doesn't show a close relationship with the rest of the subpopulations in the primary tumors, implying that only this subpopulation successfully survived and colonized a lymph node. This result seems to corroborate Filder's selection model in which primary tumor contains heterogeneous subpopulations with different abilities to metastasize [21]. Only the subpopulation that is competent for completing all steps in tumorigenesis can establish a metastasis in the second organ [21].

However, we frequently found that two subpopulations in a primary tumor show close cell linage similarity with two subpopulations in matching lymph node metastasis, respectively (Model E and Model F). This implies the two subpopulations of the primary tumor both successfully survive and proliferate in the metastasis. This might be understandable, if one considers the anatomical structure of lymph vessels, serving as a transport route for a metastasizing embolism [22]. More compelling evidence for the existence of heterogeneous embolic cell communities is provided by histopathological observations. Ruiter et al confirmed that intravascular micrometastases (lymphatic and blood vessels) had tissue-like textural features. Moreover, the structural and functional organization of intravascular tumor cells as a tissue has a key role in providing the optimum microenvironment for sustained malignant dissemination during circulation [23].

The above both relationships between primary tumor and lymph node metastasis support the hypothesis that lymph node metastases are derived from the accumulation of heterogeneous social tumor cells and that this heterogeneity provides more candidates for successful establishment of metastasis. When a metastatic cell community settles down in a lymph node, the heterogeneous immigrated cells can take the selective pressure in new environment. Taken together, the combined effects of the anatomy of the lymph system and the selective pressures might lead to the establishment of complex lymph node metastasis similar to the primary tumor.

The "normal" breast tissue samples that were collected far from the primary tumor site also exhibited some genomic aberrations. Notably, some aberrations are common between these four samples, so most of the subpopulations in them were clustered together. There could be several types of relationship between the "normal" aberrant subpopulations of tissues and the corresponding primary tumors: They could develop totally independently from their primary tumor. In some cases they might have contributed to the development of a tumor subpopulation (e.g. N49-P1/P2 and M49-P1). Alternatively, one subpopulation in "normal" tissue showing a close relationship with one subpopulation in the primary tumor might suggest that it was derived from that primary tumor.

We also need to be aware that the above models deduced by the heterogeneity model are established on the basis of the assumption of the mathematical model of tumor heterogeneity. The heterogeneity model assumes that new subpopulations in tumor inherit essential genomic aberrations from the previous subpopulation. In addition, they acquire more genomic aberrations (more losses and gains *de novo *or more extensive of aberrations in the previous loci) based on the previous subpopulation. The process of this development is assumed to be nonreversible. Since CGH techniques only detect copy number aberrations, the heterogeneity model does not consider DNA-dosage balanced genomic aberrations. Further studies involving higher resolution methods and sequencing-based platforms as well as more tumor samples will increase the precision and power of tumor analyses based on tumor heterogeneity modeling.

Our heterogeneity model and findings based on this model provide a new approach in cancer research. Knowledge of the heterogeneity of tumors could deepen our understanding of the biological behavior of cancers and might confer useful insights for clinical practice.

## Competing interests

The authors declare that they have no competing interests.

## Authors' contributions

JL performed the majority of experiments of array CGH, analyzed the data and drafted the manuscript. KW and CW designed and built up the mathematic model, analyzed the data and drafted the manuscript. SL performed programming for the model. TDJ performed array CGH analysis for colon cancer samples. LB organized the collaboration, joined the discussion and modified the manuscript. All authors read and approved the final manuscript.

## Supplementary Material

Additional file 1 **The recurrent genomic aberrations in the P1 subpopulation in more than50% of the samples for each type tumor**. Click here for file

Additional file 2 **The recurrent genomic alternations in the development of P0 P1, P1 P2, and P2 P3 in more than 50% of the samples for each type tumor**. Click here for file

## References

[B1] Heppner G Yamashina K Miller B Miller F Tumor heterogeneity in metastasis Prog Clin Biol Res 1986 212 45 59 3714732

[B2] Daidone MG Silvestrini R Valentinis B Persici P Mezzanotte G Squicciarini P Orefice S Salvadori B Proliferative activity of primary breast cancer and of synchronous lymph node metastases evaluated by [3H]-thymidine labelling index Cell Tissue Kinet 1990 23 401 408 2245439 10.1111/j.1365-2184.1990.tb01133.x

[B3] Dexter DL Leith JT Tumor heterogeneity and drug resistance J Clin Oncol 1986 4 244 257 3944607 10.1200/JCO.1986.4.2.244

[B4] Aubele M Werner M Heterogeneity in breast cancer and the problem of relevance of findings Anal Cell Pathol 1999 19 53 58 10746434 10.1155/1999/960923 PMC4617520

[B5] Jensen LH Cruger DG Lindebjerg J Byriel L Bruun-Petersen G Jakobsen A Laser microdissection and microsatellite analysis of colorectal adenocarcinomas Anticancer Res 2006 26 2069 2074 16827146

[B6] Andersen CL Wiuf C Kruhoffer M Korsgaard M Laurberg S Orntoft TF Frequent occurrence of uniparental disomy in colorectal cancer Carcinogenesis 2007 28 38 48 10.1093/carcin/bgl086 16774939

[B7] Lips EH van Eijk R de Graaf EJ Doornebosch PG de Miranda NF Oosting J Karsten T Eilers PH Tollenaar RA van Wezel T Morreau H Progression and tumor heterogeneity analysis in early rectal cancer Clin Cancer Res 2008 14 772 781 10.1158/1078-0432.CCR-07-2052 18245538

[B8] Wang K Li J Li S Bolund L Wiuf C Estimation of tumor heterogeneity using CGH array data BMC Bioinformatics 2009 10 12 10.1186/1471-2105-10-12 19134174 PMC2640360

[B9] Parkin DM Bray F Ferlay J Pisani P Global cancer statistics, 2002 CA Cancer J Clin 2005 55 74 108 10.3322/canjclin.55.2.74 15761078

[B10] Heppner GH Miller BE Tumor heterogeneity: biological implications and therapeutic consequences Cancer Metastasis Rev 1983 2 5 23 10.1007/BF00046903 6616442

[B11] Fearon ER Vogelstein B A genetic model for colorectal tumorigenesis Cell 1990 61 759 767 10.1016/0092-8674(90)90186-I 2188735

[B12] Clausen OP Aass HC Beigi M Purdie KJ Proby CM Brown VL Mattingsdal M Micci F Kolvraa S Bolund L Deangelis PM Are keratoacanthomas variants of squamous cell carcinomas? A comparison of chromosomal aberrations by comparative genomic hybridization J Invest Dermatol 2006 126 2308 2315 10.1038/sj.jid.5700375 16728973 PMC2423224

[B13] Li J Wang K Li S Timmermans-Wielenga V Rank F Wiuf C Zhang X Yang H Bolund L DNA copy number aberrations in breast cancer by array comparative genomic hybridization Genomics Proteomics Bioinformatics 2009 7 13 24 10.1016/S1672-0229(08)60029-7 19591788 PMC5054221

[B14] Li J Gromov P Gromova I Moreira JM Timmermans-Wielenga V Rank F Wang K Li S Li H Wiuf C Omics-based profiling of carcinoma of the breast and matched regional lymph node metastasis Proteomics 2008 8 5038 5052 10.1002/pmic.200800303 19003862

[B15] Jensen T Analysis of sporadic colorectal cancer using array-based comparative genomic hybridization PhD thesis 2007

[B16] de Hoon MJ Imoto S Nolan J Miyano S Open source clustering software Bioinformatics 2004 20 1453 1454 10.1093/bioinformatics/bth078 14871861

[B17] Spandidos DA Wilkie NM Malignant transformation of early passage rodent cells by a single mutated human oncogene Nature 1984 310 469 475 10.1038/310469a0 6462235

[B18] Stratton MR Campbell PJ Futreal PA The cancer genome Nature 2009 458 719 724 10.1038/nature07943 19360079 PMC2821689

[B19] Celis JE Gromov P Cabezon T Moreira JM Ambartsumian N Sandelin K Rank F Gromova I Proteomic characterization of the interstitial fluid perfusing the breast tumor microenvironment: a novel resource for biomarker and therapeutic target discovery Mol Cell Proteomics 2004 3 327 344 10.1074/mcp.M400009-MCP200 14754989

[B20] Haddad R Ogilvie RT Croitoru M Muniz V Gryfe R Pollet A Shanmugathasan P Fitzgerald T Law CH Hanna SS Microsatellite instability as a prognostic factor in resected colorectal cancer liver metastases Ann Surg Oncol 2004 11 977 982 10.1245/ASO.2004.03.585 15525826

[B21] Fidler IJ Kripke ML Metastasis results from preexisting variant cells within a malignant tumor Science 1977 197 893 895 10.1126/science.887927 887927

[B22] Swartz MA Skobe M Lymphatic function, lymphangiogenesis, and cancer metastasis Microsc Res Tech 2001 55 92 99 10.1002/jemt.1160 11596154

[B23] Ruiter DJ van Krieken JH van Muijen GN de Waal RM Tumour metastasis: is tissue an issue? Lancet Oncol 2001 2 109 112 10.1016/S1470-2045(00)00229-1 11905791

